# Shaping a Subwavelength Needle with Ultra-long Focal Length by Focusing Azimuthally Polarized Light

**DOI:** 10.1038/srep09977

**Published:** 2015-05-06

**Authors:** Fei Qin, Kun Huang, Jianfeng Wu, Jiao Jiao, Xiangang Luo, Chengwei Qiu, Minghui Hong

**Affiliations:** 1 Department of Electrical and Computer Engineering, National University of Singapore, 4 Engineering Drive 3, 117576, Singapore; 2 Singapore Synchrotron Light Source (SSLS), National University of Singapore, 5 Research Link, 117603, Singapore; 3 State Key Laboratory of Optical Technologies on Nano-Fabrication and Micro-Engineering, Institute of Optics and Electronics, Chinese Academy of Science, P. O. Box 350, Chengdu 610209, China

## Abstract

Flat optics, which could planarize and miniaturize the traditional optical elements, possesses the features of extremely low profile and high integration for advanced manipulation of light. Here we proposed and experimentally demonstrated a planar metalens to realize an ultra-long focal length of ~240λ with a large depth of focus (DOF) of ~12λ, under the illumination of azimuthally polarized beam with vortical phase at 633 nm. Equally important is that such a flat lens could stably keep a lateral subwavelength width of 0.42λ to 0.49λ along the needle-like focal region. It exhibits one-order improvement in the focal length compared to the traditional focal lengths of 20~30λ of flat lens, under the criterion of having subwavelength focusing spot. The ultra-long focal length ensures sufficient space for subsequent characterization behind the lens in practical industry setups, while subwavelength cross section and large DOF enable high resolution in transverse imaging and nanolithography and high tolerance in axial positioning in the meantime. Such planar metalens with those simultaneous advantages is prepared by laser pattern generator rather than focused ion beam, which makes the mass production possible.

Breaking the Abbe diffraction limit is a hot topic for its significant influence on practical applications, such as high density optical data storage, super-resolution imaging and ultra-precise manipulation^1^,^2^,^3^. Metamaterials with negative refractive index can achieve subwavelength focusing and imaging such as superlens and hyperlens^3^,^4^,^5^. As the primal experimental demonstration of the super-resolution imaging by negative refractive index in the quasi-static limit^6^, Luo et al experimentally observed the deep-subwavelength interference effect of surface plasmon with a silver grating in 2004, and higher resolution in 2008 by utilizing the hyperbolic dispersion and associated plasmonic filtering property of metal-dielectric layers^7^,^8^,^9^. But all the above super-resolution imaging demonstrated by metamaterials only formed in near field since the evanescent wave need to be involved. Another method to suppress the focal spot is to use the novel polarization of light such as cylindrical vector beams, and a smaller focal spot can be expected if more light energy is concentrated in the marginal part of beams^10^,^11^. It has been demonstrated that the focusing spot with strong longitudinal components can be obtained when the radially polarized (RP) beam is focused by a high numerical-aperture (NA) spherical lens. By contrast, the azimuthally polarized (AP) beam has a doughnut-shaped focal spot with transverse polarization at the focal plane, which is not suitable for nano-focusing and imaging. Recently, it has been reported that this doughnut spot can be changed into a significantly sharper focal spot when a vortical phase encoded on the azimuthally polarized beam^11^,^12^,^13^,^14^,^15^, which shows the intriguing prospect in practical applications due to its sub-wavelength lateral spot size and purely transverse electric field^16^,^17^,^18^,^19^. Quickly, this azimuthally polarized beam with vortical phase (APV) was then applied in solid immersion lens system for achieving a sub-wavelength focusing spot because of its immunity to the presence of the medium interface, and was able to maintain this spot for several wavelengths after passing through interface^20^, which is superior to other polarizations.

On the other hand, the planarization of traditional optical elements has been another emerging field applied in nano-photonics, and planar focusing devices have been demonstrated during the past few years by metasurface flat lenes^21^,^22^,^23^,^24^, binary optics^25^,^26^, and SOL^27^,^28^,^29^,^30^ etc. Metasurface based flat lenses can realizes the focusing phenomenon by ultrathin array of subwavelength-spaced resonators, but the focal spot size is difficult to break the diffraction limit. Super-oscillatory lens (SOL) can realize an arbitrarily small spot without involving the evanescent wave. Unfortunately, a very strong sidelobe is inevitably aroused outside the focal spot, which is a bad influence on the imaging quality. According to the super-oscillation criterion, the focal spot larger than 0.38λ/NA does not have significant sidelobe^31^. This means that increasing the NA of focusing lens is the most efficient method to decrease the spot without a strong sidelobe. Correspondingly, a high NA lens usually implies that the large size is required if we pursue a long focal length that is much preferred in practical applications. However, large-scaled lens with fine details is a challenging issue due to their rather complicated fabrication processes, so that the dimensions of all reported SOL are around 40 μm in diameter while suffering from a small focal length around 10 μm^27^,^32^, which is a big obstacle for the scanning process in nano-imaging if the surface roughness of target sample is large.

To address these challenges, we present a high NA planar metalens with a focal length of ~240λ under the illumination of azimuthally polarized beams with vortical phase. Transversely polarized needle with a lateral size of 0.42λ, as well as ~12λ depth of focus (DOF), is achieved. To realize it, we increase the feature size of metalens to micrometers scale, making the fabrication of this large-scaled metalens available by the laser pattern generator without the requirement of low efficiency focused-ion beam (FIB) and electron beam lithography (EBL). The transverse polarization of this sub-wavelength needle is also experimentally verified by measuring its Stokes parameters. This work paves a viable path for the industrial application of planar metalens.

## Result and discussion

**Figure 1:**
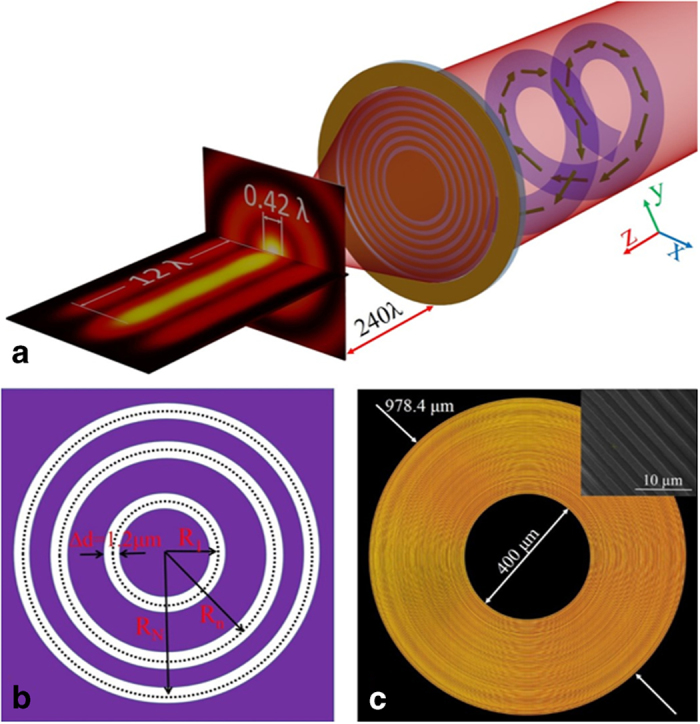
Design and effect of the planar metalens. (**a**) Sketch of shaping subwavelength needle with planar metalens induced by azimuthally polarized beam with vortical phase. The light spot on the screen in the foreground is the real focal spot taken by a CMOS camera. The vortical wavefront is denoted by the helical structure inside the incidence beam, and the polarization state is presented by the space-variant arrows. (**b**) Schematic configuration of the planar metalens. R1, Rn and R_N_ represent the radius of the 1^st^ ring, n^th^ ring and the outmost ring, respectively, and Δd represent the width of the rings which is fixed at 1.2 μm for all the rings. (**c**) Optical microscope image of the fabricated planar metalens. Inset: Sectional SEM image.

Figure 1a schematically shows the shaping of subwavelength needle with planar metalens induced by azimuthally polarized beam with vortical phase (APV). The metalens is one kind of diffractive lens depending on constructive interference of multiple beams diffracted from many transparent belts, which is different from the traditional high NA spherical lens^33^,^34^. According to vectorial Rayleigh-Sommerfeld diffraction theory^35^, for a APV beam with its electric field 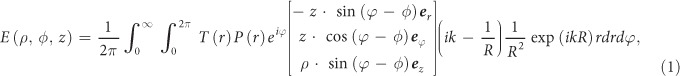
11, where *P(r)* is the amplitude factor, *r* and *φ* are the polar coordinates, at the plane z = 0 where the metalens is located, the electric field of transmitted light after the binary lens can be expressed as


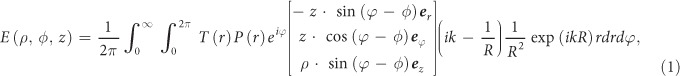


where *R*^*2*^ = *r*^*2*^ + *ρ*^*2*^ + *z*^*2*^*-2rρcos(φ-*ϕ) and *T*(*r*) is the transmission function of lens. Induced by the vortical phase of e^*iφ*^, the transmission light has a very small longitudinal *E*_z_, having a ratio of ρ/z to transverse electric field, so that the intensity of *E*_z_ is 5 orders of magnitudes smaller than that of transverse electric field for our proposed lens with a high NA, i.e. (*ρ*/*f*)^2^ = (2λ/240λ)^2^≈10^−5^, where *ρ* is valued at the order magnitude of spot size. As a result, the longitudinal component *E*_z_ is ignored in our simulations. However, if only an azimuthally polarized beam without this vortical phase is used as the illumination light, the longitudinal component *E*_*z*_ of its diffraction light is zero.

To realize both a sub-wavelength spot and ultra-long focal depth, an optimization algorithm is employed to carry out the design of the metalens by tuning the parameters of these belts, see Methods sections. Fig. 1b presents the sketch of the proposed planar metalens composed of transparent belts with a fixed width of 1.2 μm. The diameter of whole pattern is 978.4 μm, which consists of 107 concentric rings and a block with 400 μm diameter is located in the centre. A key feature of our planar metalens is their ease of fabrication, which makes mass production possible. By utilizing UV laser pattern generator, we patterned the structure on a standard chrome photo-plate, followed by a standard Cr etching, the substrate is a quartz plate with 3 inch in size. The fabrication process is much easier than low efficiency FIB which is normally used for the SOL fabrication in the previous works. Through precisely control of the fabrication process, the discrepancy of the fabricated structure with the design parameters can be controlled under 100 nm, which satisfies the condition of constructive interference when the light scatters out from the metalens. The optical microscope images of the fabricated metalens is shown in Fig. 1c, where the inset is its sectional SEM image. The detailed fabrication process is provided in Methods section.

**Figure 2:**
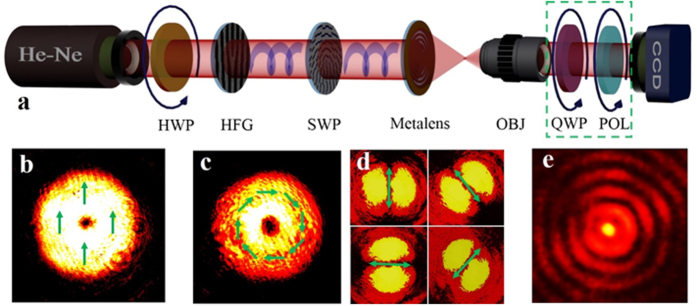
Characterization of the planar metalens focusing. (**a**) Schematic of the experimental setup. Abbreviations for optical components: HWP: half wave plate. HFG: holographic fork grating. SWP: S-waveplate. OBJ: objective lens. QWP: quarter waveplate. POL: linear polarizer. The helical structures represent the vortical phase. The two components QWP and POL inside the green dash box are only used to measure the stocks parameters. (**b**) Experimentally recorded diffraction pattern of linear polarized beam with vortical phase, which was created by the holographic fork grating. (**c**) Experimental intensity pattern of the APV beams after being modulated by the S-waveplate. The green arrows indicated the polarization states of the light beam. (**d**) The field pattern of the APV beam after being filtered by a linear polarizer at different angles. The orientations of the polarizer are indicated by the green arrows. (**e**) Intensity distribution at the focal plane which locates at 150 μm away from the metalens.

The focusing characterization was performed by a self-built microscope imaging system, as schematically shown in Fig. 2a. In this experiment, we used a He-Ne 633 nm laser with linear polarization, which direction can be tuned by a half-wave plate. The APV beam was obtained by making incidence light pass through a holographic fork grating and S-waveplate consequently. The holographic fork grating is designed by ourselves and fabricated by the same technique for the planar metalens. After travelling through a holographic fork grating, light with linear polarization was imprinted with the required helical phase of *e*^*iφ*^ and a doughnut shape field distribution, as shown in Fig. 2b. To convert the linearly vortex beam into an APV beam, a S-waveplate was adopted after the holographic fork grating. The S-waveplate is a product based on the unique laser nano-structuring technique^36^. This S-waveplate having space-variant nanostructures can convert a linearly polarized beam into an azimuthally polarized state when the direction of linear polarization is matched with the working axis of S-waveplate, as shown in Fig. 2c. We checked its polarization state by using a linear polarizer and shown its dumbbell shape intensity distributions in Fig. 2d, which experimentally confirms a well-defined azimuthally polarized beam. A super-resolution focal spot, created by the planar metalens, was recorded by a high NA objective lens combined with a CMOS camera, which is in principle similar to the dual-mode microscope commonly used for the testing of super-oscillatory lens^27^,^28^,^32^. The detail characterization process is discussed in the Methods section.

**Figure 3:**
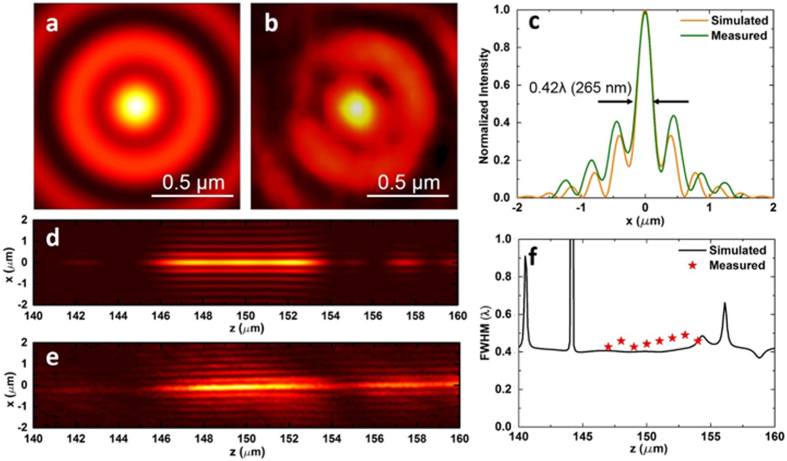
Theoretical and experimental results of the focusing property by planar metalens. (**a**) Simulated intensity distribution at the focal plane which is located at the propagation distance of z = 150 μm. (**b**) Experimental recorded intensity distribution at the focal plane. (**c**) Line-scan profiles along the diagonal across the focal spot for the simulated result (orange line) and the measured result (green line). Simulated (**d**) and measured (**e**) intensity distributions along the propagation distance ranging from 140 μm to 160 μm. (**f**) FWHM of the focal spot in the propagation direction, the black solid line represents the theoretical results and the red stars depict the experimental results with 1 μm step. False-colour map indicates the normalized intensity.

Three important parameters to evaluate a planar lens are: 1) focal spot size determining its focusing capacity; 2) focal length responsible for its working distance; 3) depth of focus governing the tolerance in practical applications. The simulated and measured intensity distributions at the focal plane of 150 μm away from the metalens are depicted in Fig. 3a and 3b. Both of their line intensity profiles across the center of focal spot are shown in Fig. 3c, which shows good agreement between simulated and measured results. The tiny discrepancy might comes from the experimental error during the characterization. The full width at half maximun (FWHM) of the experimental spot size is about 0.42λ (265 nm) in air (Fig. 3c), indicating a super-resolution spot, in contrast to Abbe diffraction limit given by this metalens is λ/2NA≈330 nm, where NA = 0.95 for our metalens. On the other hand, according to the super-oscillation criterion *R*_s_ = 0.38λ/NA≈0.4λ, this spot is not super-oscillatory, which is deliberately designed for avoiding the high sidelobe. However, our measured spot size is closely approaching this super-oscillation limitation. As a result, the central focal spot dominates the intensity at the focal plane, having a ratio of 2.5:1 between the high intensity central beam spot and the first side-lobe ring, which is similar with 3:1 in simulation results. Comparing with that of a superoscillatory lens based on the destructive inteference, the spot size of our metalens is much preferred for a better imaging quality in practical applications. In addition, the focal spot is located at the ~240λ (150 μm) away from the metalens, which is a distinctive advantage to facilitate the scanning imaging process for various kinds of samples. Besides the subwavelength focal spot, a long optical needle will be convenient to tolerate the operation error. To show this needle, the theorectical and experimental results of the intensity in the *x*-*z* plane are given in Fig. 3d and 3e. Apparently, the field distribution does not change remarkably ranging from 147 μm to 154 μm along the propagation direction. It means the depth of focus (DOF) about 12λ (7 μm) is formed, which is similar to that 15λ (6 μm) at λ = 405 nm of the reported works^27^. Both DOFs have a significant improvement compared with that (2λ/NA^2^≈2.5λ) of the traditional spherical lens^37^. It should be noted the slightly tilt in the experimetal *x-z* pattern comes from the mechanical drift duing the test scaning process. The theoretical and experimental FWHM values of the cross-section fields along the optical needle are depicted in Fig. 3f, clearly shows the super-resolution capability within a very long range. The size of experimental focal spot varies from 0.42λ to 0.49λ within the optical needle. Therefore, our metalens not only can push the focal length to hundreds of wavelengths, but also maintain a small focal spot and large DOF, showing a great potential in real applications.

**Figure 4:**
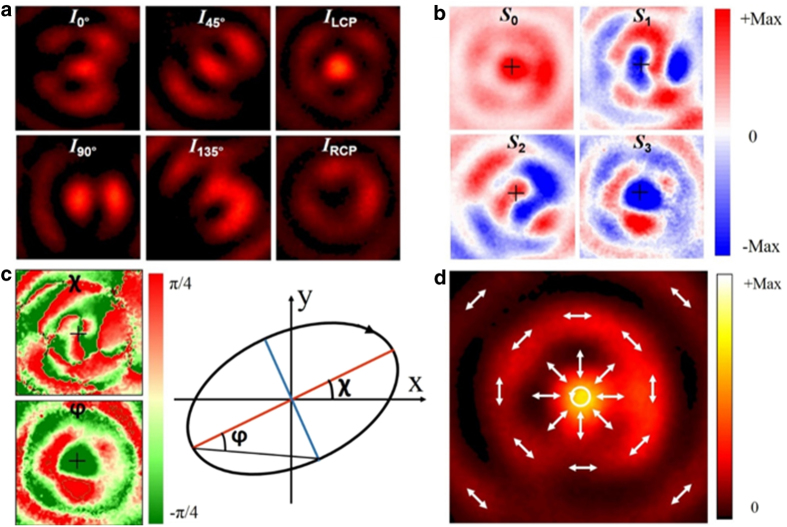
Measurement of the Stokes parameters. (**a**) Experimentally recorded intensity distribution around the focal spot region of different polarization status. (**b**) Stokes parameters derived from the experimental results by using Equation (2). (**c**) The azimuthal angle (χ) and ellipticity angle (*φ*) of the local polarization ellipse around the focal region. (**d**) Polarization profile of the focused APV beam by planar metalens.

The distinctive property of the focusing APV beam is that transverse electric component dominates the focal plane without strong longitudinal component. The polarization state of the focal spot has been theoretically presented by the previous works^12^,^20^,^38^, but its experimetal demonstration are seldom reported. Here, we used Stokes parameters to map the polarization distribution of this subwavelength spot, so that we can derive the key parameters of the polarization property, such as the shape of the ellipse, its orientation with respect to some fixed spatial axes and the direction of rotation of the ellipse^38^,^39^,^40^,^41^. For a monochromatic wave, four Stokes parameters can be experimentally measured by using the following Equation:


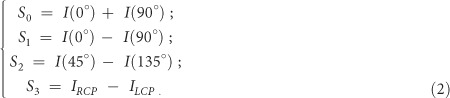


where *I(α)* is the intensity of the light polarized in the direction *α* to the *x* axis, and *I*_*RCP*_ and *I*_*LCP*_ are the intensities of right circular and left circular polarized light, respectively. The measurement of the Stokes parameters are performed by a polarization filter which consists of one quarter-waveplate followed by a linear polarizer, as shown in Fig. 2a. The intensity profiles of the variety polarization states, as shown in Fig. 4a, are measured by rotating the polarization filter. Thus, we can easily get the the Stokes parameters around the focal spot region following Equation (2) and shown them in Fig. 4b. Its parameters *χ* and *φ*, which denote the azimuthal angle and ellipticity angle respectively, of elliptical polarization are shown in Fig. 4c. The relationship between them and Stokes parameters can be found in^38^,^40^


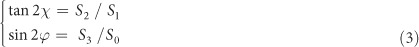


The final polarization profile of the focal spot is presented in Fig. 4d by analysing the Stokes parameters. It is clearly seen that the polarization is spatially variant. The left-handed circular polarization (LCP) is located at the center of the spot. Beyond the central LCP focal spot, the polarization gradullay changes into a radial polarization state at the first dark ring, through varing elliptical degree with some intermediate polarization states (not labeled in Fig. 4d). For the outter sidelobe, its polarization state still remains azimuthally polarized state. This resuls is similar to the theoretical results given by the previous works^12^,^20^.

**Figure 5:**
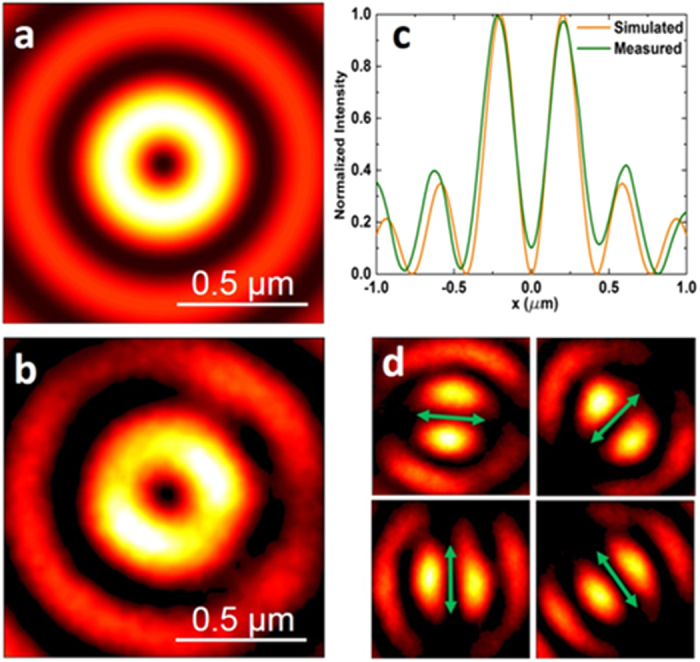
Focusing property of the azimuthally polarized beam by planar metalens. (**a**) Simulated intensity distribution at the focal plane. (**b**) Experimental recorded intensity distribution at the focal plane. (**c**) Line-scanning profiles along the diagonal across the focal spot for the simulated (orange line) and measured (green line) results. (**d**) The intensity distributions under variety collection polarization states. The transmission orientation of the polarizer is indicated by green arrows. False-colour map indicates the relative intensity.

To unveil the role of the vortical phase, a control experiment was performed by illuminating the planar metalens with a normally incident azimuthally polarized beam without vortical phase. A linearly polarized light was allowed to pass through the S-waveplate to create the azimuthlly polarization. Respondingly, as shown in Fig. 5a and 5b, both the theorectical and measured results indicated that the intensity distribution at the focal plane is a doughnut pattern, showing that a destructive interference happens at the focal plane. Their line-scanning intensity distributions along the diagonal across the focal pattern are depicted in Fig. 5c, which shows a nearly perfect consistency. The polarization property of the focal spot is also revealed by rotating the linear polarizer around the beam axis as shown in Fig. 5d, which indicates that its polarization remains to be azimuthally polarized. This shows that the vortical phase is responsible for the constructive interference, leading to a tight focusing spot with a subwavelength size.

As we known, the utilization efficiency of the incidence light is an important parameter to evaluate any optical elements for applications, especially for the aspect of integration circuit. Our metalens has the apparant advantages because the focal spot is formed by the constructive interference, in contrast with the SOL focusing which is formed by the destructive interference. In addition, the feature size of our structures is larger than the working wavelength, which should definitely increases the diffraction light intensity involved in the focusing effect. The utilization efficiency of the incidence light should be further enhanced by making the metalens by phase masks instead of amplitude masks. In addtion, The FWHM of the focal spot we demonstrated is around 0.42λ, which is similar to reported results^27^. The absolute value of focal spot will be smaller while illumination light with shorter wavelength is used, and can be further reduced by performing the characterizaion inside high refractive index environments.

## Conclusion

In summary, we designed and experimentally demonstrated the shaping subwavelength needle with planar metalens induced by azimuthally polarized beam with vortical phase (APV). The focal spot in size 0.42λ (265 nm), without strong sidelobe, is obtained. The length of the optical needle is around 12λ (7 μm). The focal length is up to around 240λ (150 μm), which is one order larger than previous works. This is a distinctive advantage for the practical applications such as nano-imaging. The polarization state of the focal region is experimentally analyzed by measuring the Stokes parameters. Our structures were fabricated by laser pattern generator, which makes the large size patterns and mass production possible. The realization of super-resolution focusing of APV beam by planar metalens is of particular relevance, as it will be unquestionably beneficial to realize functional flat optics.

## Methods

### Design and optimization

All the simulations in this paper were carried out by using Equation (1). In our simulation, the electric field of incident APV beam has a Laguerre-Gaussian (LG) distribution of 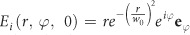, where *w*_0_ = 337.8 μm is obtained by using the curve fitting of the measured intensity at the plane of metalens. It is because light diffracting from a fork grating is a LG beam with a vortical phase. The design of the planar metalens is implemented by using the particle swarm optimization algorithm. Considering that the width of every ring in this lens is fixed at Δ*d* = 1.2 μm, we only need to determine the central radius *R*_n_ of every belt for finishing the lens design. In our optimization process, we used a particle population of 20 in one iteration and about 5000 iterations were carried out to finish the design of the proposed metalens. Its optimized parameters are shown in Table 1.

### Samples Fabrication

The planar metalens and the holographic fork grating were fabricated by a UV laser pattern generator (Heidelberg, DWL-66FS). The structures were patterned on a standard chrome photoplates, which is 530 nm AZ1518 photoresist coated on 100 nm Cr film, substrate is quartz plate with 3 inch in size (Nanofilm, 3*3*0.06-QZ-LRC-5M-1518-5K). The design structures were created by Autocad with dxf format, and then transferred into LIC format to be read by laser pattern generator. The diameter of the metalens pattern is 978.4 μm, which consists of 107 concentric annuli each with a fixed width at 1.2 μm. During the patterning, the substrate was held down on the stage by vacuum and scanned in the x-y plane with 40 nm step. The defocus value and intensity setpoint are 2500 and 40, respectively. 10% transmission filter is placed in front of the writer head. After the patterning, a standard develop process was used to develop samples in AZ 400 K developer at room temperature for 30 sec, (followed by rinsing with running DI water for 60 s to wash off the residual developer thoroughly, and then dry the sample by N_2_ blowing). Then the standard Cr etching process was carried by placing the plate in chromium etchant 1020AC and agitated gently at room temperature for 90 seconds, (followed by rinsing with DI water to wash the etchant away the plate thoroughly and dried by N_2_ blowing). After inspected the fabrication quality by optical microscope, we strip the mask for 5 minutes in Aceton to remove the AZ photoresist at room temperature with gentle agitation. The fabricated structures were inspected and imaged using scanning electron microscope with an accelerating voltage of 5 kV (NOVA NanoSEM 230).

### Characterization

The experimental setup is a self-built imaging system. A schematic of the test principle is shown in Fig. 2a. The light source used in the experiments is a low power He-He linear polarized laser (MellesGriot, 25-LHP-925-230). A half-wave plate was utilized to rotate the polarization orientation of the laser beam after the light emits out from the laser, then illuminated on a holographic fork grating to create a vortical phase with topological charge L = 1. The 1^st^ diffraction order of the transmission beams was selected, and we used a plano-convex lens with 200 mm focal length (not presented in Fig. 2a) to collimate the vortex beam because the small size (400 μm in diameter) of the fork grating leads to a slight divergence of the generated vortex beam, which creates a doughnut shape cylindrical light beam with 1 mm diameter, as shown in Fig. 2b. A S-waveplate (Altechna, RPC-632-04) was used in the following to convert the vortical phase encoded linear polarized beam into a azimuthally polarized beam with vortical phase (APV) (Fig. 2c and 2d), and finally illuminated on the sample from the substrate side with the field pattern exactly overlapping the planar metalens. A high magnification and high NA objective (Olympus, LMPlan Apo 150X, NA0.9 BD) was used to collect the diffraction pattern and then imaged by a high resolution CMOS camera (The imaging source, DMK 72BUC02, 2592 × 1944 pixel) to obtain the *x-y* field pattern . The validity of this technique has been verified and often used in the published papers. For the purpose of the calibration to the magnification of the imaging system, we used the structure as the reference plane and got an image firstly during each measurement. Since the structure parameters were already known in the design and confirmed by the SEM inspection before the characterization, so we could know the denotative size of each pixel of the CMOS camera to the image plane. Then we got the size of focal spot by counting the pixel quantity when we located the focal spot on the imaging plane. A high precision 1D stage was used to scan the objective lens in z direction with a step size of 100 nm, and then map the electric field in the longitudinal plane to get the *x-z* cross-section of the intensity distribution. For the measurement of stocks parameters, we inserted a polarization filter, which consists of one quarter wave plate followed by a linear polarizer, in between the objective lens and the collection camera, as shown in Fig. 2a. The intensity distribution under variety collection polarization states can be obtained through rotating the quarter wave plate and the polarizer around the beam axis. For the four linear polarization variants, *I(0°), I(90°), I(45°), I(135°)*, they can be obtained by only rotating the orientation of the polarizer at different angles, where *I(α)* is the intensity of the light polarized in the direction *α* to the x axis. The intensity distribution of right circular and left circular polarized components,*I*_*RCP*_ and *I*_*LCP*_, were collected by rotating the transmission axis of linear polarizer at 45° and 135°, while fixing the quarter wave plate along *x* axis. Same exposure time of the CMOS camera was used during the recording for variety polarization states. Then the stocks parameters were derived from the polarization patterns by Equation (2). The focusing effect induced by the azimuthally polarized beam was characterized by removing the holographic fork grating from the imaging system.

## Additional Information

**How to cite this article**: Qin, F. *et al*. Shaping a Subwavelength Needle with Ultra-long Focal Length by Focusing Azimuthally Polarized Light. *Sci. Rep.* **5**, 09977; doi: 10.1038/srep09977 (2015).

## Figures and Tables

**Table 1:** Geometry of the metalens.

**n**	***R***_**n**_ **(μm)**	**n**	***R***_**n**_ **(μm)**	**n**	***R***_**n**_ **(μm)**	**n**	***R***_**n**_ **(μm)**
1	**200**	28	**273.2**	55	**347.5**	82	**421.1**
2	**202.6**	29	**276**	56	**350.4**	83	**423.9**
3	**205.4**	30	**278.7**	57	**353.1**	84	**426.5**
4	**208.1**	31	**281.5**	58	**355.8**	85	**429.1**
5	**210.6**	32	**284.1**	59	**358.6**	86	**431.7**
6	**213.3**	33	**286.7**	60	**361.4**	87	**434.4**
7	**215.9**	34	**289.5**	61	**364.1**	88	**437**
8	**218.5**	35	**292.2**	62	**366.7**	89	**439.8**
9	**221.3**	36	**294.8**	63	**369.5**	90	**442.6**
10	**223.9**	37	**297.5**	64	**372.3**	91	**445.4**
11	**226.5**	38	**300.2**	65	**375.1**	92	**447.9**
12	**229.1**	39	**302.9**	66	**377.6**	93	**450.7**
13	**231.9**	40	**305.6**	67	**380.3**	94	**453.4**
14	**234.7**	41	**308.6**	68	**383.1**	95	**456.1**
15	**237.4**	42	**311.4**	69	**385.8**	96	**458.8**
16	**240.5**	43	**314.2**	70	**388.5**	97	**461.5**
17	**243.2**	44	**317**	71	**391.2**	98	**464.3**
18	**245.8**	45	**319.9**	72	**394**	99	**466.9**
19	**248.5**	46	**322.6**	73	**396.6**	100	**469.5**
20	**251.7**	47	**325.4**	74	**399.3**	101	**472.3**
21	**254.4**	48	**328.1**	75	**402**	102	**475.1**
22	**257.1**	49	**330.9**	76	**404.7**	103	**477.8**
23	**259.7**	50	**333.8**	77	**407.3**	104	**480.5**
24	**262.4**	51	**336.5**	78	**410.1**	105	**483.3**
25	**265**	52	**339.5**	79	**412.8**	106	**485.9**
26	**267.7**	53	**342.1**	80	**415.6**	107	**488.6**
27	**270.5**	54	**344.7**	81	**418.3**		
